# Social and spatial conflict drive resident aggression toward outsiders in a group-living fish

**DOI:** 10.1093/beheco/arab045

**Published:** 2021-05-25

**Authors:** Jakob Gübel, Aneesh P H Bose, Alex Jordan

**Affiliations:** Department of Collective Behaviour, Max Planck Institute of Animal Behavior, Universitätsstraße 10, 78464, Konstanz, Germany; Centre for the Advanced Study of Collective Behaviour, University of Konstanz, Universitätsstraße 10, 78464, Konstanz, Germany; Department of Biology, University of Konstanz, Universitätsstraße 10, 78464, Konstanz, Germany; Department of Collective Behaviour, Max Planck Institute of Animal Behavior, Universitätsstraße 10, 78464, Konstanz, Germany; Centre for the Advanced Study of Collective Behaviour, University of Konstanz, Universitätsstraße 10, 78464, Konstanz, Germany; Department of Biology, University of Konstanz, Universitätsstraße 10, 78464, Konstanz, Germany; Department of Collective Behaviour, Max Planck Institute of Animal Behavior, Universitätsstraße 10, 78464, Konstanz, Germany; Centre for the Advanced Study of Collective Behaviour, University of Konstanz, Universitätsstraße 10, 78464, Konstanz, Germany; Department of Biology, University of Konstanz, Universitätsstraße 10, 78464, Konstanz, Germany

**Keywords:** cichlid, conflict, group, resource, social, spatial

## Abstract

Group-living animals often experience within-group competition for resources like shelter and space, as well as for social status. Because of this conflict, residents may aggressively resist joining attempts by new members. Here, we asked whether different forms of competition mediate this response, specifically competition over 1) shelter, 2) spatial position within groups, and 3) social or sexual roles. We performed experiments on wild groups of *Neolamprologus multifasciatus* cichlids in Lake Tanganyika, either increasing or decreasing the number of shelters (empty snail shells) within their territories. We predicted that increases in resource abundance would reduce conflict and lower the aggression of residents toward presented conspecifics, while decreases in resources would increase aggression. We explored the effects of social conflict and spatial arrangement by introducing same or opposite sex conspecifics, at greater or lesser distances from resident subterritories. We found that changing the abundance of shells had no detectable effect on the responses of residents to presented conspecifics. Rather, aggression was strongly sex-dependent, with male residents almost exclusively aggressing presented males, and female residents almost exclusively aggressing presented females. For females, this aggression was influenced by the spatial distances between the presented conspecific and the resident female subterritory, with aggression scaling with proximity. In contrast, presentation distance did not influence resident males, which were aggressive to all presented males regardless of location. Overall, our results show that group residents respond to presented conspecifics differently depending on the type of competitive threat these potential joiners pose.

## Teaser

Group living is associated with numerous challenges, including how to share limited space and resources among group members, and how to divide social roles. These challenges generate conflict among group members and outsiders. We find that changing within-group resource abundance does not affect responses, but that aggression is highest when residents and outsiders are of the same sex, and for females, when the outsider is in close proximity to her subterritory.

## INTRODUCTION

Group living can increase competition for shared resources, because individuals living in groups, by definition, inhabit the same place at the same time. This competition can lead to conflict among group members over shared resources like food (Barton et al. 1996) or shelter (Ford and Swearer 2013), as well as social and sexual roles (Bergmüller and Taborsky 2010). Although co-habitation can lead to increased competition over mutually required resources (e.g., in social spiders, Majer et al. 2015; Majer et al. 2018), this conflict can be ameliorated by changes in overall resource abundance, which in turn may reduce barriers to group living, for example, normally aggressive solitary spiders become more docile and aggregative in areas of high prey availability (Rypstra 1986). Competition for resources within groups can also be reduced through divergent patterns of space use; in red-winged blackbirds, *Agelaius phoeniceus*, females defend subterritories against other females within the male’s broader territory (Nero 1956a; Nero 1956b; Beletsky 1983) and although the ecological resources required by these females may be shared, separation in space reduces the realized conflict. Finally, competition for social position, in which individuals occupy similar social (or sexual) roles and therefore compete most strongly with one another, can lead to intra-group conflict, a dynamic frequently examined in the context of the polygyny threshold model (Grønstøl 2018; Jungwirth and Johnstone 2019). Indeed, we here utilize the conceptual framework provided by the polygyny threshold model, which largely focuses on decisions of prospecting females when choosing among territorial males of varying quality, resource abundance, and degree of current polygyny, assuming that resources on a male’s territory are shareable and depreciable among settling females. However, spatial structure of the territory might reduce the shareability of resources and hence mediate polygyny threshold conflicts between residents and potential joiners.

Competitive interactions within a group also have consequences for resident responses to potential new group joiners, especially if the joiner would fulfil an already-present role in the group. A conflict of interest between residents and non-group members occurs because for residents additional group members may increase within-group competition, while providing only marginal benefits (Higashi and Yamamura 1993), whereas solitary individuals may be willing to accept increased competition for resources to gain the benefits of group living (e.g., increased foraging success, Krebs 1974; Morgan 1988, or reduction in predation risk, Pitcher et al. 1988; Krause and Ruxton 2002). Sibly group size (Sibly 1983) describes the phenomenon that animal groups commonly contain more members than a predicted optimal group size, as solitary individuals gain more from joining groups even if overall group function is decreased. However, these groups are typically “free-entry,” that is, existing group members do not, or cannot, exclude new members from joining. For many animal groups, however, especially those with more sophisticated social systems, membership and hierarchy are stable over time and space (Krause and Ruxton 2002). In these systems, residents exert influence over group membership, and may attempt to exclude new members, creating “restricted entry” groups (Stephens et al. 2005; Jordan et al. 2010). Residents may resist joining attempts by individuals based on familiarity (Payne et al. 1991; Radford 2005), but resistance toward non-residents may also depend on the degree of competition with existing residents, for example in damselfish, responses to unfamiliar individuals depend strongly on size (Jordan et al. 2010). Responses of residents to new group members therefore depend on a number of factors, including existing within-group competition, resource availability, and potential risk of competition with new members.

Here, we examine three sources of potential conflict—resource conflict, space conflict, and social conflict—and explore how these interact and shape the responses of group residents toward unfamiliar conspecifics. We performed field experiments on naturally occurring wild groups of the cichlid fish *Neolamprologus multifasciatus*, one of the shell-dwelling Lamprologine cichlids, in Lake Tanganyika (Lein and Jordan 2021). Stable breeding groups consist of one to three males, one to five adult females and multiple juveniles, and the fish excavate empty gastropod shells from the sediment that they use as brood chambers and shelters from predation (Jordan et al. 2016). The widespread digging activity of these fish can create large swaths of uncovered shells, or so-called “shell beds” on the lake floor (Salzburger et al. 2014). The territories of *N. multifasciatus* can be visually identified as distinct depressions in the sand in which collections of empty shells are found (Rossiter 1993; Konings 1998). While the dominant male defends the entire territory against intruders, females occupy subterritories that they defend, including against other females both from within and outside their own group (Kohler 1998). A low number of shells in a female’s subterritory has been linked to elevated female conflict and female emigration (Schradin and Lamprecht 2002), and increasing the availability of shells in a territory attracts more interest from reproductive females in the population but also attracts more aggression from rival males (Jordan et al. 2016). Thus, altering the abundance of gastropod shells within an *N. multifasciatus* territory provides a direct manipulation of ecological resource abundance, with both reductions and increases of shells having been shown to affect intra-group dynamics.

We combined manipulations of shell number in territories with presentations of foreign male or female conspecifics, presenting these unfamiliar individuals at differing distances from each subterritory. In a related Lamprologine species, dispersal into new groups is preceded by a prospecting phase, in which conspecifics sequentially visit territories prior to joining new groups (Jungwirth et al. 2015), and our “fish-in-a-bottle” experiments were designed to mimic these prospecting visits. Nevertheless, it is possible these individuals were perceived as territory intruders or even as predators on fry, an interpretation we also examine. We then recorded the aggressive responses of residents toward the presented fish, as well as aggression among the resident fish themselves during the presentation. We predicted that decreasing the abundance of shells in a territory would increase resource conflict and therefore increase aggressive responses of all group members toward presented conspecifics. In contrast, increasing the overall abundance of shells was predicted to decrease resident aggression toward presented individuals, because ecological resource competition would be reduced. A reduction in resident female aggression toward outsider females may facilitate joining by new females, which has been observed when adding shells to groups in previous studies in this population (Jordan et al. 2016). Because spatial position can influence access to resources and food, as well as mediate exposure to predation, we also predicted that residents would show spatially dependent responses to presented individuals, with more aggression toward more closely presented conspecifics. Furthermore, we predicted the aggression amongst the residents themselves might also be mediated by their own relative proximities to the presented conspecifics. Finally, we expected a strong effect of social (specifically sexual) role, with males being most aggressive to presented males, and females being most aggressive to presented females.

## METHODS

### Field work and social group selection

**Figure 1:**
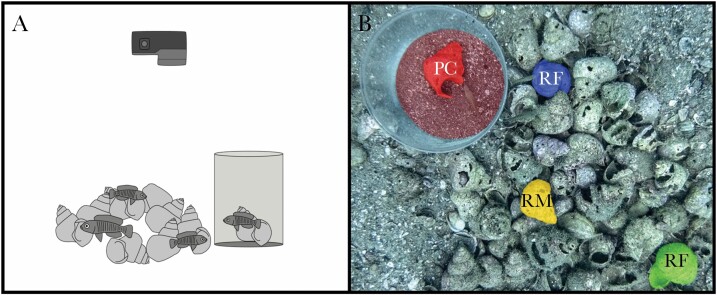
(A) Schematic of experimental set-up. Top-mounted video camera above wild *Neolamprologus multifasciatus* group, including the cylinder in which foreign conspecifics were presented to the resident fish. (B) Still-frame from video footage. Colors indicate the home shells of the presented conspecific (PC, red), the resident male (RM, yellow), and the two resident females (RF, blue and green), as well as the presentation cylinder (light red).

Field work took place off the southern shore of Chikonde Village, Mutondwe Island, Zambia (8°42′49.0′′S 31°07′22.9′′E) in October and November 2018. This field site contains a large breeding population of *N. multifasciatus*, located on a shell bed at a depth of 9–11 m. Groups typically contain 1–3 males and 0–5 females, along with numerous juveniles (Jordan et al. 2016), and while there is no pronounced sexual dimorphism in coloration, males are larger than females (males 24.5 mm median standard length; females 19.0 mm median standard length; Jordan et al., in review), and males are typically more aggressive than females (Jordan et al. 2016). Relatedness structure within and among groups is unclear, but it has been suggested that females are the dispersing sex and males may inherit their natal territories (Kohler 1998). Ten social groups were selected while SCUBA diving, each consisting of one adult male, two adult females, and several juveniles. Top-mounted video cameras (GoPro Hero 6) were installed 55 cm above each group (Figure 1A). After cameras were set up, an observer (JG) remained motionless from a distance of approximately two meters away from the group and made a count of the number of visible gastropod shells in each group’s territory and the home shell of each individual (the shell into which it retreated when threatened). The sex composition of each group was also determined based on their social behavior and relative body sizes, an approach that was confirmed by dissecting fish after similar field observations in a parallel study conducted concurrently in the same population (A.B. and A.J., personal observations). The standard length (SL) of each resident fish and total territory area (cm^2^) were subsequently measured in Adobe Photoshop CC from still frames of the video recordings taken by the cameras in which a ruler was placed for reference.

### Competition and resident response experiments

We applied three experimental treatments to the ten selected social groups, using a within-groups repeated-measures design. In the “shell addition” treatment, the number of shells in the focal territory was increased by as close to 20% as possible by taking empty, available shells from the wider shell bed environment (these shells were removed again immediately after the trial). In the “shell subtraction” treatment, ~20% of the shells in the territory were removed and temporarily placed 2 m away from the focal group (these shells were returned to their original locations in the territory immediately after the trial). In the control treatment, ~20% of the visible shells in the territory were taken away and then immediately returned to their original places. These 20% shell manipulations were spatially concentrated in areas of the males’ territories where 1) there was no fish’s home shell, such that the home shell of a resident fish was never disturbed during the handling process, and 2) there was a sufficient number of shells present to be taken or supplemented. Previous studies (Jordan et al. 2016) suggest that manipulation of more than this ratio of shells increases risk of territory takeover by larger heterospecifics, so a ratio of ~20% was the maximum manipulation we considered reasonable for this study. The groups were all given 24 h between each treatment and the following observation recording. Immediately after each observation, the groups were given their next treatment and again allowed 24 h before their subsequent observation.

In each trial, a conspecific from a distant territory (at least 20 m away) was taken, along with its home shell, and placed in a transparent plexiglass cylinder (8 cm diameter). The cylinder was placed on the edge of the focal territory, within 2 cm of one of the peripheral shells, and interactions among the resident and presented fish were recorded (Figure 1B). Both males and females were chosen to be presented, and each focal *N. multifasciatus* group received all three shell manipulation treatments in the presence of a presented female and also a presented male. The male presentations and the female presentations each took place in short succession to one another during the observation phases of each experimental treatment (counterbalancing for order). Thus, every social group experienced three shell manipulation treatments (one treatment per day, in randomized order), and the ensuing behavioral interactions between residents and presented fish were observed for each treatment. Trials took place between 9:00 and 14:00. The presented fish were returned to their home territories after completion of their trials and not used again in any further experimental trials.

Recording was started after the placement of the cylinder containing the presentation fish. The presented fish emerged from its shell while within the cylinder 85 ± 87 s (mean ± SD, range = 15–423) after placing it on the territory edge. These presentations elicited appreciable levels of aggression toward the presented fish but also aggression amongst the resident fish themselves, which had previously showed little or no intra-group aggression. All aggressive interactions were scored for a 10-min period. Behavior was scored manually using the software BORIS (Friard and Gamba 2016). Because manipulations were visually apparent, the scorer (J.G.) could not be blind to treatment. Behaviors were scored using the ethogram presented in Table 1 and pooled into one count of aggression. Note that frontal displays were rare in our observations, and sometimes difficult to accurately assess from the top-down field video footage, and were therefore not included in our counts of aggression. Although we scored all aggressive acts occurring amongst the resident fish, aggression by resident females toward resident males was also exceptionally rare; across all of our 10-min trials, resident females aggressed against their males a total of 19 times, a sample insufficient to draw statistical inferences from. Furthermore, aggression between resident females was also rare, occurring only 35 times and only in seven field videos. Our statistical comparisons of within-group aggression across experimental treatments therefore focus on resident male versus female aggression. Lastly, we measured the distances between each resident fish’s home shell and the presented cylinder for each trial using the Adobe Photoshop CC.

**Table 1:** Ethogram of behaviors scored from videos of *Neolamprologus multifasciatus* in the field. These behaviors represent a subset of the complete behavioral repertoire of *N. multifasciatus* and only include contest behaviors observed during experimental manipulations

Behavior	Description
Aggression	
Lateral display	Focal fish positions its body laterally with another fish and adopts a rigid body. Often accompanied by focal fish thrashing its caudal fin toward the opponent.
Bite	Focal fish swims quickly toward opponent making contact, or is repelled by the plexiglass barrier.
Lunge	Focal fish accelerates quickly toward other fish, but stops short before making contact with the opponent or plexiglass barrier.
Other	
Shell hiding	Focal fish remains fully within the gastropod shell and thus cannot interact with other fish.

### Statistical analysis

All statistical analyses were conducted in R (v. 3.6.2, R Core Team 2019). To test whether shell manipulations influenced the aggression by the resident fish toward the presented fish, we fit a generalized linear mixed effects model (GLMM) assuming a quasi-Poisson error distribution with a log link function (using the “nbinom1” family from the glmmTMB R package, Brooks et al. 2017). We included the counts of aggressive acts by each resident fish toward the presented fish as the response variable, as well as treatment (three-level categorical variable: control, shell addition, shell subtraction), sex of the presented fish (two-level categorical variable: male, female), and sex of the resident fish as predictor variables, along with each of their pairwise interaction terms. In addition, we included the distance between the resident fish’s home shell and the presented fish (cm, but scaled so that mean = 0, SD = 1) as another predictor variable along with its interaction with sex of the resident fish. Finally, we also included the order in which the shell manipulation treatments were given to account for potential order effects. We included a random intercept of fish ID nested within territory ID to account for nonindependence of responses (because multiple *N. multifasciatus* individuals per group were repeatedly tested across treatments). As a model offset term, we included the cumulative time durations over which both the resident fish and the presented fish were outside their shells and thus had the opportunity to interact (log-transformed). We tested whether inclusion of the interaction terms significantly improved model fit based on a likelihood ratio test (LRT), and if not, we omitted them. We used the “emmeans” R package (Lenth 2020) to make further comparisons using the Tukey method.

Next, we tested whether resident male-to-resident female aggression varied with the sex of the presented fish. To do this, we fit a GLMM assuming a quasi-Poisson error distribution (“nbinom1” from glmmTMB). We included the counts of aggressive actions by the resident male toward resident females as the response variable. Treatment and sex of the presented fish were included as predictor variables, and we tested whether to include their interaction term based on a LRT (as above). As above, we also included the order in which the shell manipulation treatments were given. We included a random intercept of female ID nested within male ID and also a model offset term to account for differing time windows when both the male and each resident female were out of their shells and thus had the opportunity to interact.

Finally, we focused only on the scenario when the presented fish was female, and we tested whether resident male aggression toward his resident females was disproportionately directed toward the resident females that were currently closer to the presented female. Here, we fit a binomial GLMM using the “logit” link function. We included a binary response variable indicating whether or not the attacked resident female was the closer of the two females. We also included treatment as a predictor variable as well as a random intercept of female ID nested within male ID.

## RESULTS

Resident females were on average (± SD) 21.3 ± 1.5 mm SL (range = 18.2–24.4 mm), while resident dominant males were 29.6 ± 1.4 mm (range = 27.9–31.4 mm). Presented females were 20.7 ± 1.4 mm SL (range = 18.3–23.5 mm), while the presented males were 28.9 ± 1.4 mm SL (range = 26.5–31.7 mm). Territories had an average of 30.1 ± 7.0 (range = 17–41) shells. Of the 60 total trials (10 social groups, six treatment combinations), six could not be analyzed due to technical and field logistical issues. Of the remaining 54 trials, all fish presentations elicited aggressive responses from the resident fish. Over the course of our experiment, resident females rarely aggressed against other resident females (*N* = 35 aggressive acts in total) or against their resident males (*N* = 19 aggressive acts in total). As such, these interactions were not further examined (but can be found in the uploaded data files, see Data Accessibility).

### Shell manipulations did not influence sex-specific responses to presented conspecific

**Figure 2:**
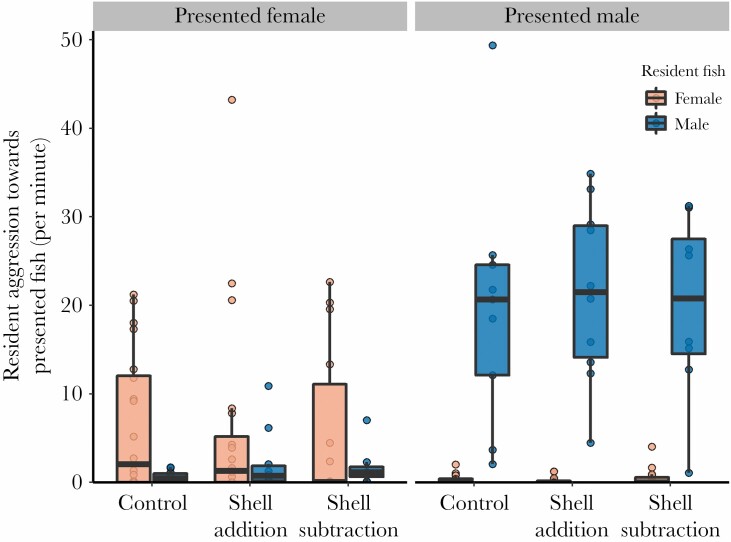
Aggression by resident fish toward the presented conspecific fish as a function of shell manipulation treatment, sex of the resident fish and sex of the presented fish. Y-axis is presented as a rate, in acts per minute, which was calculated by taking the raw behavioral counts for aggression and dividing them by the time period when both the resident aggressor and the presented fish were emerged from their shells and thus had the opportunity to interact. The boxplots overlaying the datapoints show medians (horizontal bar), the first and third quartiles (box), and the range of data within 1.5 interquartile distances above and below the interquartile (whiskers).

**Figure 3:**
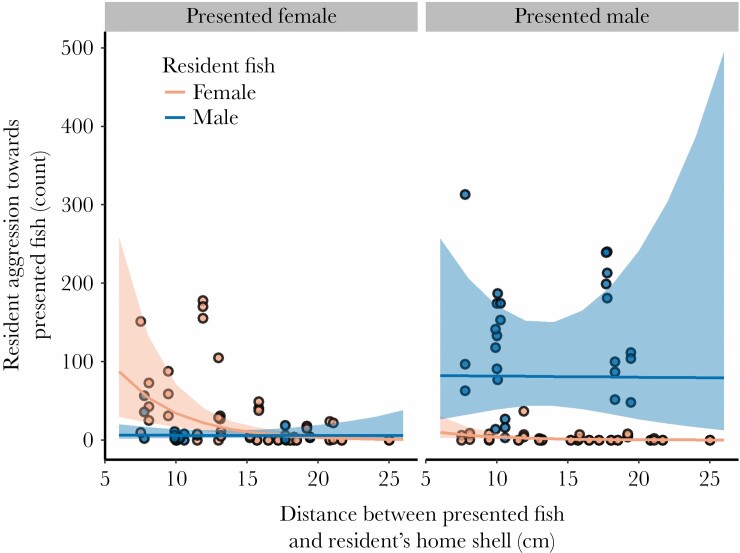
Aggression by resident fish toward the presented conspecific fish as a function of the distance separating the resident fish from the presented fish. Dots indicate the raw behavioral counts for aggression observed during each trial, while the lines and ribbons indicate the predicted fits and 95% confidence intervals from a generalized linear mixed-effects model (see Methods). Note that this model accounts for varying time windows (via a model offset) wherein both the resident aggressor and the presented fish were emerged from their shells and thus had the opportunity to interact. Model predictions were visualized using the “ggeffects” R package (Lüdecke 2018).

Shell manipulation treatment did not have a statistically significant influence on resident-presented fish aggression (Table 2, all simple emmeans contrasts between treatments were at most, est. ± SE = 0.026 ± 0.14, *t*-ratio_149_ = 0.19, *P* = 0.98). However, the aggression displayed by resident fish toward presented fish depended strongly on the sexes of both individuals (GLMM, interaction term between sex of presented fish and sex of resident fish, Table 2; Figure 2). We broke this interaction down with the use of “emmeans” contrasts. Averaging over treatments, resident female aggression was not significantly higher than resident male aggression when the presented fish was female (est. ± SE = 0.60 ± 0.46, *t*-ratio_149_ = 1.33, *P* = 0.19), but resident male aggression was significantly higher than resident female aggression when the presented fish was male (est. ± SE = 4.21 ± 0.47, *t*-ratio_149_ = 8.90, *P* < 0.0001, Figure 2). Resident male aggression was significantly higher when the presented fish was male rather than female (est. ± SE = 2.62 ± 0.24, *t*-ratio_149_ = 11.06, *P* < 0.0001), and resident female aggression was similarly higher when the presented fish was female rather than male (est. ± SE = 2.19 ± 0.25, *t*-ratio_149_ = 8.82, *P* < 0.0001, Figure 2). Finally, the distance between the resident fish’s home shell and the presented fish strongly affected the level of aggression by the resident fish, but also depended on the sex of the resident (GLMM interaction term, Table 2). In particular, increasing distance significantly reduced the aggression given by resident females toward the presented fish but this effect was not as strong for resident males (Figure 3).

**Table 2:** Statistical output for generalized linear mixed effects model (*N* = 161) examining the effects of shell manipulation treatment and spatial positioning of fish on the territory on the aggression of resident fish toward presented fish

Conditional model	Estimate ± SE	z	*P*
Intercept	−3.12 ± 0.32	−9.64	**<0.001**
Shell manipulation treatment			
Control treatment (reference category)			
Shell addition treatment	−0.026 ± 0.14	−0.19	0.85
Shell subtraction treatment	−0.0089 ± 0.15	0.058	0.95
Sex of presented fish			
Female (reference category)			
Male	−2.19 ± 0.25	−8.82	**<0.001**
Sex of resident fish			
Female (reference category)			
Male	−0.60 ± 0.46	−1.33	0.19
Spatial distance on territory (scaled)	−1.10 ± 0.27	−4.04	**<0.001**
Order of treatments	−0.026 ± 0.08	−0.33	0.74
Sex of presented fish × sex of resident fish	4.81 ± 0.36	13.26	**<0.001** ^†^
Sex of resident fish × spatial distance on territory (scaled)	1.09 ± 0.42	2.59	**0.0096**

Note that females are always treated as the reference category for both sex of the resident fish and sex of the presented fish, as is the control group for the shell manipulation treatment. Random effects structure consists of a random intercept of “fish ID” nested within “territory ID.” Significant *P*-values at α = 0.05 are given in bold.

^†^This interaction term is broken down into its constituent parts in-text with “emmeans” contrasts.

### Aggression by resident males toward resident females depended on sex of presented conspecific

**Figure 4:**
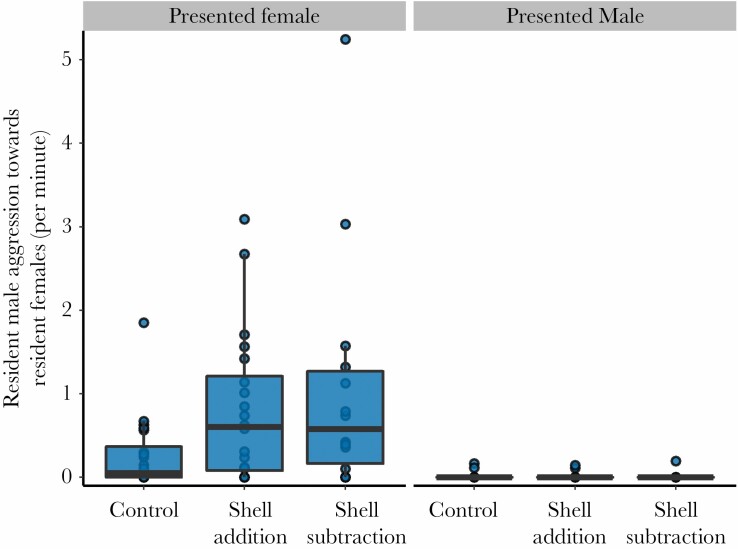
Aggression by resident males toward his own resident females as a function of shell manipulation treatment and sex of the presented fish. Y-axis is presented as a rate, in acts per minute, which was calculated by taking the raw behavioral counts for aggression and dividing them by the time period when both the resident male and his resident females were emerged from their shells and thus had the opportunity to interact. The boxplots overlaying the datapoints show medians (horizontal bar), the first and third quartiles (box), and the range of data within 1.5 interquartile distances above and below the interquartile (whiskers).

Resident males aggressed against their own females more often when the presented fish was female relative to when it was male (Table 3; Figure 4). Males also aggressed against their own females more often when shells were added to their territory relative to the control group (Table 3). In no other treatment group, comparisons did males clearly aggress more often against their own females.

**Table 3:** Statistical output for generalized linear mixed effects model (*N* = 107) examining the effects of shell manipulation treatment on the aggression of resident males toward their own resident females

Conditional model	Estimate ± SE	z	*P*
Intercept	−5.18 ± 0.48	−10.76	**<0.001**
Shell addition treatment	0.75 ± 0.34	2.24	**0.025**
Shell subtraction treatment	0.72 ± 0.38	1.92	0.056
Sex of presented fish (male)	−2.98 ± 0.48	−6.18	**<0.001**
Order of treatments	0.029 ± 0.18	0.16	0.87

Note that females are treated as the reference category for sex of the presented fish, and the control group is treated as the reference category for the shell manipulation treatment. Random effects structure consists of a random intercept of “female ID” nested within “male ID.” Significant *P*-values at α = 0.05 are given in bold.

### Resident males did not aggress more against females that were closest to the presented female

The likelihood that males would attack the closer of the two resident females (i.e., the resident female closest to the presented female) did differ among the treatment groups. Males in the control group were more likely to attack the closer of the two resident females when compared to males in the shell subtraction group (GLMM, est. ± SE = 3.29 ± 1.41, z = 2.33, *N* = 220, *P* = 0.020), but not when compared to males in the shell addition group (est. ± SE = 2.02 ± 1.19, z = 1.70, *P* = 0.089). Males in the shell addition group did not differ from the shell subtraction group in this regard (est. ± SE = 1.27 ± 0.72, z = 1.76, *P* = 0.079). However, the model intercepts for each treatment group can also be used to assess whether males are more or less likely *than chance* to attack the closer female. Examining these intercepts suggests that males were not more likely *than chance* to attack the closer of the two females in any of the treatment groups: control group (est. ± SE = 2.50 ± 1.50, z = 1.67, *P* = 0.095), shell addition group (est. ± SE = 0.48 ± 1.27, z = 0.38, *P* = 0.71), and shell subtraction group (est. ± SE = −0.79 ± 1.36, z = −0.58, *P* = 0.56).

## DISCUSSION

In wild groups of the cichlid *Neolamprologus multifasciatus*, we explored the role of conflict in shaping responses to presented unfamiliar conspecifics. Competition for food, shelter, and space are known to drive divergence in behavior and distribution across species, but can also influence intraspecific conflict and niche specialization (Bolnick 2001). Here, we extend this research and show that the competition within social groups can influence the response of residents toward intruders or potential group joiners. In contrast to our predictions, this conflict was not strongly influenced by group-wide ecological (shelter) competition, but rather by interindividual spatial and social relationships. When we manipulated the overall resource abundance in groups (either increasing or decreasing shell number by 20%), we did not detect clear changes in the patterns of aggression toward presented conspecifics for either sex of resident. Our results suggest that, in this species at least, contest behavior is maximally expressed regardless of territory quality, or that aggressive responses occur without any assessment of territory quality, as may be predicted for example under no assessment models (Arnott and Elwood 2009). Alternatively, our treatments may have been too conservative to elicit a behavioral response, or applied for too short a time period to have been perceived by residents. In a previous study employing similar techniques, the addition of a similar proportion of shells to territories resulted in higher rates of female immigration into those territories (Jordan et al. 2016), but this effect was observed over a period of weeks rather than the shorter period of this study.

We did however observe an effect of the sex of the presented conspecific on resident responses; male residents almost exclusively aggressed presented males, while female residents almost exclusively aggressed presented females. This almost binary effect suggests that social conflict, here likely driven by potential conflict over access to mates (for males) and conflict over territory space and resources (for females), has a strong effect on resident responses to presented conspecifics. This finding supports our conclusion that presented individuals were not perceived as territory intruders or fry predators, in which case we would expect uniform aggression by all residents to both sexes of presented fish. Instead, it appears presented fish were perceived as potential group-joiners, and responded to depending on the degree of conflict a joining event would generate with residents of either sex. These dynamics are well-explored by the polygyny threshold model, which has long been used to shed light on demographic structure in polygynous groups (Orians 1969; Davies 1989; Grønstøl 2018). From a male perspective, joining females may lead to direct fitness gains associated with access to a new breeding partner (Wade 1979). As we observed here, resident males showed relatively less resistance when the presented fish was female while the opposite was true for resident females, and this is likely due to a mis-alignment of interests between the sexes of resident individuals as a function of divergent payoffs between the sexes (Davies 1989; Shuster 2009). Similarly, while resident males aggressed heavily against presented males, resident females showed little aggression. Such a response by females may either be a consequence of the inability of the smaller females to fight with larger males, or due to some benefit accrued to females by the presence of additional males. It is possible that females may benefit from the additional territory defense provided by males, or that these males are potential breeding partners for resident females. Interestingly, our observations of high aggression by resident females toward presented females, was accompanied by an increase in aggression from dominant males toward their own resident females during these presentations. Such responses have previously been interpreted as male attempts to “police” or dissuade the efforts of resident female(s) in repelling potential joiner females (Grønstøl 2018), and a similar pattern of behavior, termed “peace-keeping,” has been previously documented both in this species (Schradin and Lamprecht 2000) and in the closely related *Lamprologus ocellatus* (Walter and Trillmich 1994). Although we detected increased aggression from dominant males toward resident females during female presentations, the males did not preferentially attack the closer of the two resident females to the presented fish (based on our interpretations of the model intercepts, see Results), despite the closer female being expected to be the most aggressive. Thus, male peace-keeping behavior, at least in our sample, may simply be elevated overall and not necessarily directed toward specific females, nor those more likely to aggress potential joiners. Taken together, we interpret the strong sex-specific response to joiners, as well as the intra-group sexual conflict, to be a consequence of the mis-alignment of interests between the sexes in groups; residents suffer costs from the incorporation of same-sex joiners, but accrue benefits when opposite-sex individuals join.

The spatial separation between a resident’s home shell and that of the presented fish had a strong, sex-specific influence on fight dynamics. Resident females were highly aggressive toward females that were presented in close proximity to their home shell. Dominant males on the other hand, showed heightened aggression toward presented males with no statistically clear reduction in aggression with distance across the spatial scales that we tested. This pattern is likely a consequence of the spatial structuring of *N. multifasciatus* territories, in which males traverse the entire territory, and even move beyond the territory borders, whereas females are largely confined to smaller subterritories (Schradin and Lamprecht 2002). It is important to note that overall territory size did not covary with the distances measured between the presentation cylinder and the residents’ home shells; that is, the distances over which interactions took place between residents and presented fish could be relatively far or short regardless of the size of the focal territory (post-hoc, linear mixed effects model, controlling for territory ID, showed no effect of territory area, *P* = 0.96, on distances between resident home shell and presented fish). A female presented in close proximity to a resident female’s home shell may be perceived as a more threatening competitor for her subterritory resources or for other factors associated with her specific location on the territory, such as exposure to predation, access to food, or maintenance costs. For example, groups of *N. multifasciatus* feed on passing plankton borne on the current, and for many species of fish that feed in the same way, dominant individuals defend positions relative to the prevailing current direction, thereby ensuring priority access to food, often aggressively displacing subordinates (Coates 1980; Nakano 1995; Webster and Hixon 2000; Whiteman and Côté 2004). Additionally, the spatial position of an individual *N. multifasciatus* subterritory may mediate predation risk, for instance due to selfish-herd type effects (Hamilton 1971; Krause 1993). In a related species, *Neolamprologus pulcher*, individuals prefer to occupy vacant territories that are more central in communities, potentially due to differences in predation risk (Heg et al. 2008). At a smaller spatial scale, certain locations within a territory may be preferred by females, who defend these positions against rival females. Additionally, position in the broader community may affect information transfer (Rosenthal et al. 2015; Rodriguez-Santiago et al. 2020), and particularly in large colonies of *N. multifasciatus*, nearby groups may act as an early warning system of threats (Büscher 1986). Yet certain spatial positions may also be disadvantageous; in *N. multifasciatus* groups individuals continually perform maintenance of their territories, removing sand from within their subterritory and depositing it into adjacent groups. As such, certain locations that are continually covered with sand by rival groups may be less preferred due to the increased maintenance costs in these locations (Kohler 1998). Our findings that female resident responses to presented females were location dependent suggests one or more of these factors may play a role, and future work could fruitfully explore the relative effect of each.

A less straightforward result was that experimental additions and subtractions of shells were accompanied by additional aggression by the dominant male toward his resident females, relative to the control group, specifically when unfamiliar females were presented. This effect was statistically significant in the shell addition treatment but not significant for the shell subtraction treatment (Table 3). It is possible that when shell abundance is altered within a territory, the borders of the subterritories that comprise the wider territory shift or become uncertain. In such an event, within-group aggression may occur to re-establish boundaries, perhaps necessitating increased policing from the dominant male, an idea that would require further testing in the future. Further work may also examine whether manipulating a higher proportion of shells per group, or doing so over a longer period of time, causes greater differences (either reduction or increase) in aggressive responses to presented conspecifics. It may also be necessary to determine the individual quality of added or removed shells (for example, the degree of structural intactness; Bose et al. 2020) as these attributes of shelters may alter perceived territory quality, and hence behavioral responses. Finally, future manipulations should account for the distribution of female subterritories (which was only revealed in subsequent analyses) as inadvertent biases in manipulations could influence female responses, for example by adding or subtracting shells from one female more than another. However, if some females did experience stronger manipulations than others, then under such a scenario we would predict that variance among the resident female aggressive responses would increase; specifically, that resident female-joiner female aggression would be more variable in the shell addition and removal treatments than in the control treatment, which we did not observe.

Our findings have direct relevance to two aspects of the polygyny threshold model. First is that although we only observed male to female peace-keeping, in stable group-living polygynandrous systems, peace-keeping could potentially also extend to resident males aggressing toward prospecting male joiners and resident females interfering in these conflicts. Closely related cichlid species, in which females are larger, and in which females benefit from multiple males in their group, for example in the cichlid *Julidochromis transcriptus* (Kohda et al. 2009; Li et al. 2015) would be ideal candidates to test these predictions. Conceptually, this reversed scenario has also received scarce research attention to date. In general, resource polygyny (and polygynandry) can underlie both intra- and inter-sexual conflict, and all the possible pairwise interactions between males, females, residents, and joiners are important to consider when empirically examining the polygyny threshold model. Second, recent extensions of the polygyny threshold model have included considerations of variation in female competitive strength and relatedness among female competitors (Grønstøl 2003; Grønstøl et al. 2015; Grønstøl 2018) and our findings of strong spatial effects on female–female aggression suggest more generally that spatial effects too can help to explain polygynous settlement patterns and conflicts across taxa.

Overall, we find that social and spatial competition affect resident responses to presented conspecifics in *N. multifasciatus*, but the effects of changing resource abundance are less clear. Responses of residents to unknown individuals appear to be a function of the potential for conflict if these presented individuals were to join the group, and we find that this conflict should be examined from multiple perspectives accounting for the effects of resources, space, sexual, and social conflict. We find evidence that within groups, interests are not aligned, and that resident males and resident females may have divergent interests in accepting new group members. Our study demonstrates that both resident-joiner, as well as resident–resident conflict depend on both social competition and spatial territory structure, a result with relevance to many other group-living animals.

### Ethics approval

Data collection was performed using unattended underwater cameras and caused minimal disturbance to the animals. This work adhered to the ASAB/ABS Guidelines for the Use of Animals in Research and field work was carried out with the permission of the Fisheries Department of Zambia under a study permit issued by the government of Zambia (SP 008735) and a memorandum of understanding (MOU 101/14/11), and adhered to the regulations of the Zambian Prevention of Cruelty to Animals act. The study species is listed “Least Concern” on the IUCN Red List of Threatened Species.

## FUNDING

JG and AJ were supported by The Max Planck Society. AB was supported by an Alexander von Humboldt Research Fellowship. This research was funded by the Deutsche Forschungsgemeinschaft (DFG, German Research Foundation) under Germany’s Excellence Strategy - EXC 2117 - 422037984 and by the Department of Collective Behaviour, Max Planck Institute of Animal Behavior.

## Data Availability

Analyses reported in this article can be reproduced using the data provided by Guebel et al. (2021).
